# Psychological and Related Factors Influencing Adherence to Biologic Therapies in Asthma: A Scoping Review

**DOI:** 10.1007/s11882-025-01242-5

**Published:** 2025-12-17

**Authors:** Valentina Poletti, Eleonora Volpato

**Affiliations:** 1 Department of Psychology, Università Cattolica del Sacro Cuore, CAP, Via Nirone, 15, Milan, 20123 Italy; 2 IRCCS Fondazione Don Carlo Gnocchi, Milan, Italy

**Keywords:** Adherence, Asthma, Biologic therapy, Scoping review

## Abstract

**Purpose of Review:**

This scoping review aimed to explore psychological and related biopsychosocial factors influencing adherence to biologic therapies among individuals with asthma. Despite the proven efficacy of biologics for severe type 2 inflammation, real-world adherence remains variable. The review sought to identify individual, clinical, and contextual determinants that facilitate or hinder adherence behaviors.

**Recent Findings:**

A systematic search of PubMed, Scopus, and APA PsycINFO identified 14 studies published between 2018 and 2025. Most were observational or retrospective and conducted in the United States. Adherence rates were generally moderate to high but varied across biologics. Systemic barriers included high out-of-pocket costs, complex insurance procedures, and limited access to specialist care. Clinically, patients with milder symptoms or low perceived treatment benefit were more likely to discontinue. Psychological barriers—such as fear of injections, illness denial, depression, and stigma—were recurrent yet understudied. Facilitators included perceived efficacy, emotional reassurance, shared decision-making, and supportive treatment settings.

**Summary:**

Adherence to biologic therapies in asthma reflects a multidimensional interplay between systemic, clinical, and psychological influences. Integrating psychological assessment, patient education, and system-level support is essential to sustain long-term engagement. Future research should address psychological mechanisms and develop tailored interventions to enhance adherence and improve clinical outcomes.

**Supplementary Information:**

The online version contains supplementary material available at 10.1007/s11882-025-01242-5.

## Introduction

Asthma is a chronic disease of the airways, characterized by inflammation, structural remodeling, and functional abnormalities that lead to bronchial hyperresponsiveness and reversible airflow limitation [1]. While standard therapies, such as combinations of inhaled corticosteroids (ICS), long-acting β2-agonists (LABAs), and leukotriene receptor antagonists, achieve good symptom control in most asthma individuals, about 5–10% continue to have poorly controlled disease [1, 2]. Severe or treatment-resistant asthma affects only a small proportion of patients but contributes disproportionately to the overall burden of the disease in terms of cost, complications, and mortality [3]. Moreover, comorbidities such as rhinitis, gastroesophageal reflux, obesity, and obstructive sleep apnea (OSA) can further complicate management in these individuals, who often experience greater impairment in daily functioning and an increased risk of anxiety and depression [3, 4].

In recent years, the understanding of asthma as a heterogeneous condition has led to a shift in treatment paradigms. Rather than a one-size-fits-all approach, asthma is now recognized as comprising distinct phenotypes and endotypes—subtypes characterized by shared clinical features and underlying inflammatory mechanisms. One of the most relevant distinctions is between T2-high (type 2 inflammation) and T2-low asthma [2]. Approximately 50–80% of patients with asthma present with a T2-high endotype, which may be allergic (IgE-mediated) or eosinophilic (IL-5-mediated), and is driven by cytokines such as interleukin-4 (IL-4), IL-5, and IL-13 [3].

Biologic therapies have emerged as a transformative option for patients with severe T2-high asthma who are unresponsive to high-dose ICS plus additional controllers [2]. These treatments consist primarily of monoclonal antibodies that selectively target key molecules in the inflammatory cascade [2]. For example:

- Omalizumab is an anti-IgE monoclonal antibody approved for allergic asthma; it binds to circulating IgE and prevents its interaction with receptors on mast cells and basophils.
- Mepolizumab, Reslizumab, and Benralizumab target IL-5 or its receptor, thereby reducing eosinophil survival and activity.
- Dupilumab inhibits IL-4 and IL-13 signaling by blocking the shared IL-4Rα receptor subunit, modulating both allergic and eosinophilic inflammation [1, 3, 5].

Clinical trials and real-world evidence have shown that these biologics can significantly reduce exacerbation rates, improve lung function, enhance quality of life, lower the reliance on systemic corticosteroids in appropriately selected patients [5, 6]. These agents offer a more targeted and personalized approach to asthma management, particularly in individuals with elevated biomarkers, such as blood eosinophils, serum IgE, or fractional exhaled nitric oxide (FeNO) [8].

However, the clinical efficacy of biologic therapies relies heavily on consistent, long-term adherence. Although their pharmacological effectiveness is well-established, adherence remains suboptimal in some patients, potentially undermining treatment outcomes [9]. As with other chronic therapies, adherence to biologics is influenced by a complex interplay of clinical, social, and psychological factors. Patients’ beliefs about their illness [10], emotional coping styles [11], and levels of psychological distress [12] can all significantly affect adherence behavior, either positively or negatively. Unlike inhaled therapies, biologic treatments for severe asthma involve infrequent but parenteral administration, often in specialized care settings and under continuous medical supervision. These differences may shape patients’ experiences and adherence patterns in distinct ways. For instance, while inhaled medications require daily self-management and routine integration into daily life, biologics demand procedural acceptance, confidence in injection safety, and sustained motivation to attend scheduled administrations.

Despite the growing integration of biologics into asthma treatment algorithms, the role of psychological determinants of adherence remains under-investigated. The traditional biomedical model often overlooks these factors, even though evidence increasingly highlights their pivotal role in shaping treatment engagement and outcomes. A deeper understanding of the psychological barriers and facilitators to adherence could guide the development of more effective, individualized interventions [13]. By synthesizing the available literature, the review emphasizes the central role of biopsychosocial determinants in shaping adherence to biologic therapies in asthma. Through this analysis, it aims to elucidate key underlying mechanisms and highlight the value of integrating psychological perspectives to enhance adherence and improve long-term outcomes for patients with asthma.

## Materials and methods

Given the complexity and multidimensionality of psychological influences on adherence—as well as the fragmented and heterogeneous nature of existing evidence—a scoping review represents an appropriate and timely methodology to comprehensively map current knowledge and identify critical gaps. Unlike systematic reviews, this approach offers the advantage of enabling a comprehensive and nuanced exploration of complex, multidimensional constructs that may not be adequately captured by narrowly defined research questions or homogenous study designs [14]. Guided by a deliberately broad research question rooted in the Population, Concept, and Context (PCC) framework [15], this review explores: *What are the psychological and related factors (Concept) that influence adherence to biologic therapies (Context) in patients with asthma (Population)?* Specifically, it seeks to identify individual-level variables that either facilitate or hinder adherence behaviors in this population.

This scoping review was conducted in accordance with the methodological framework proposed by Arksey and O’Malley (2005) and reported following the PRISMA-ScR guidelines [15–17].

### Search Strategy

To address this question, we conducted a methodical search of literature using electronic databases including PubMed, Scopus, and APA PsycINFO. Keywords included combinations of terms such as *asthma*, *biologics*, *adherence*, *adherence to treatment*. Articles were included if they (1) focused on patients with asthma undergoing biologic treatment, and (2) discussed adherence-related outcomes in connection with psychological or behavioral factors.

The complete search strategy for each database is provided in Additional File 1.

### Study Selection Process

All studies published up to May 2025 were considered for inclusion. The review encompassed review papers, clinical trials, and editorials. Specifically, editorials and conceptual articles were included for their theoretical value in framing biopsychosocial determinants but were excluded from critical appraisal. The search was limited to peer-reviewed publications. Grey literature, conference abstracts, and non–peer-reviewed reports were excluded to ensure the methodological rigor and comparability of included studies.

Only full-text papers written in English and available in their final published form were eligible. There were no limitations on author nationality, or funding resources. Searches were conducted between April 1 and May 31, 2025.

**Fig. 1:**
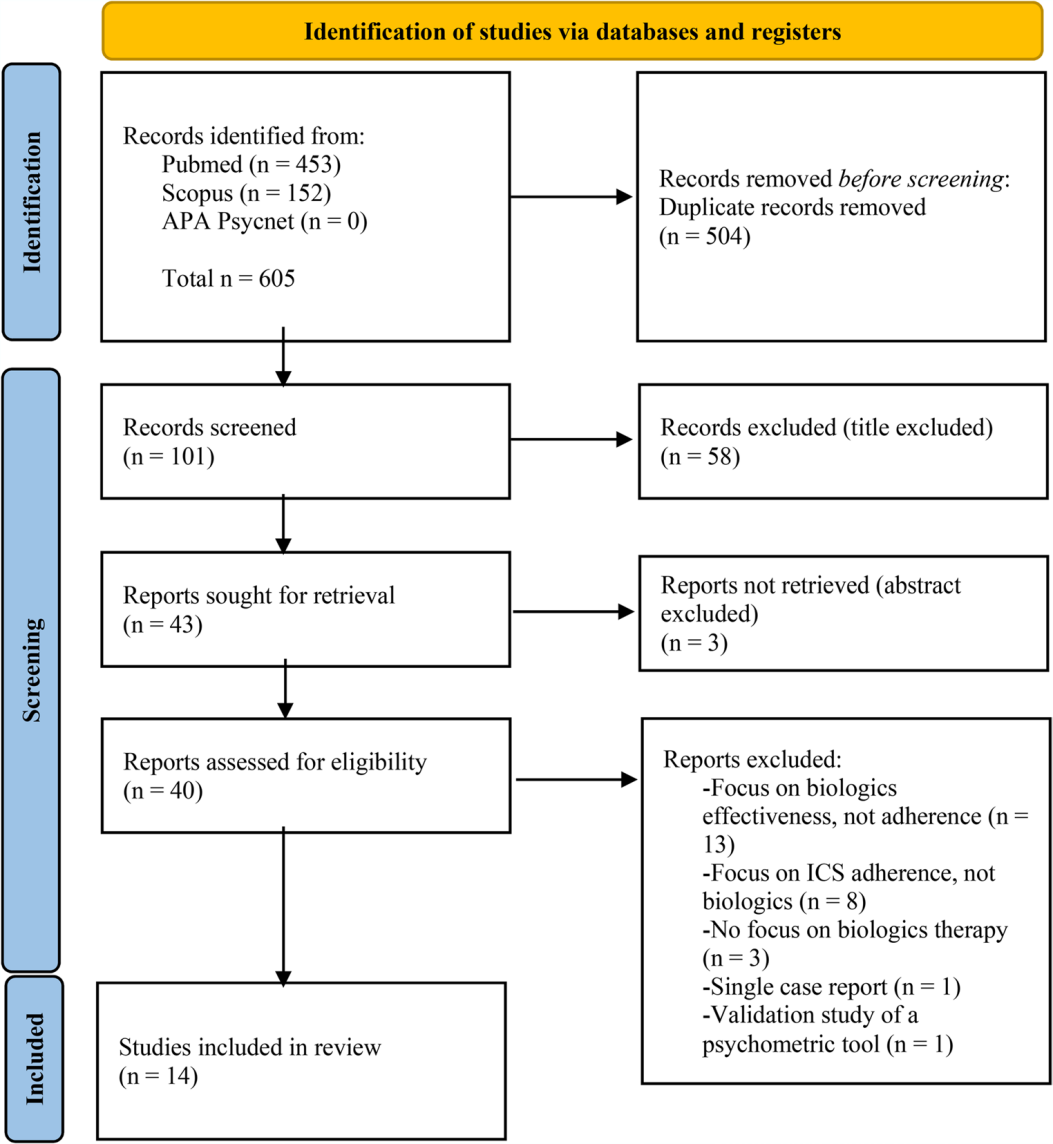
Flowchart of study selection process

Figure 1 presents the PRISMA-ScR flow diagram outlining the study selection process.

### Data Extraction

For each included study, the following data were systematically extracted and recorded using a standardized data charting form: (1) Author(s), country, and year of publication; (2) Study design; (3) Participant characteristics, including asthma severity classification and total sample size; (4) Age distribution and sex ratio of the sample; (5) Type(s) of biologic therapy administered; (6) Reported barriers to adherence and facilitators of adherence to biologic treatments; (7) Method used to assess adherence (e.g., Proportion of Days Covered, pharmacy refill data, or survey); and (8) Adherence rates or indicators, when available. When relevant, additional qualitative or contextual information related to adherence behaviors or clinical characteristics (e.g., use of maintenance oral corticosteroids, comorbidities such as nasal polyposis, or reasons for switching/stopping treatment) were also collected. Two independent reviewers screened titles and abstracts. Full-text articles were reviewed in duplicate. An informal consultation with clinical experts in asthma management was conducted to validate the relevance of emerging themes.

### Critical Appraisal

Given the heterogeneity in study designs, populations, and adherence assessment methods across the included studies, a formal risk of bias assessment using standardized tools was not conducted. Instead, a narrative and contextual appraisal was performed to evaluate the methodological transparency and relevance of each source. Particular attention was paid to the clarity of study aims, sample characteristics, adherence measurement methods [e.g., use of objective indices such as Proportion of Days Covered (PDC) versus self-report], and reporting of psychological or contextual variables relevant to adherence behavior. Studies employing real-world data and validated adherence metrics were considered more methodologically robust than those relying exclusively on narrative descriptions or unstructured surveys. Editorials and conceptual papers were excluded from quality rating but were included for their theoretical relevance in shaping a biopsychosocial understanding of adherence. In line with the exploratory aims of this scoping review, no studies were excluded based on methodological quality, but variations in rigor were noted and considered in the interpretation of findings.

## Results

A total of 12 (without considering 2 editorials) articles were included, published between 2018 and 2025, with the majority conducted in the United States (*n* = 11, 70%). The relatively recent publication dates reflect the progressive approval and clinical integration of biologic therapies for asthma. Omalizumab, the first biologic therapy for asthma, was approved in 2003, followed by Mepolizumab in 2015, Benralizumab in 2017, Dupilumab in 2018, and Tezepelumab in 2021. Table 1 reports all the included studies’ characteristics, considering author(s), country, year of publication, study design, asthma severity, sample size, age, male-female ratio, biologic type, barriers and facilitators of adherence, adherence evaluation system, and adherence rate details.

**Table 1:** Studies’ characteristics

Author(s)	Country	Year	Study Design	Participants	Asthma Severity	Sample Size (*n*)	Sample Age	Male/Female Ratio	Biologic Type	Barriers to Adherence	Facilitators of Adherence	Adherence evaluation system	Adherence Rate Details
Bender et al.	USA	2022	Retrospective, observational real life study	Asthma patients	154 (30%) GINA Step 2 11 (2%) GINA Step 3 104 (21%) GINA Step 5 126 (25%) GINA Step 6	506	< 18: 18 (3%) 18–30: 48 (9%) 31–45: 130 (26%) 46–60: 227 (45%) > 61: 85 (17%)	157 (31%)/349 (69%)	omalizumab, mepolizumab, reslizumab, benralizumab, dupilumab	Mild-to-moderate asthma; Absence of chronic oral corticosteroid (OCS) use;	Chronic use of OCS (≥ 90 days); High disease severity (GINA Step 5) with established maintenance therapy	Proportion of Days Covered (PDC)	Of 403 (total: 506) patients with maintenance medication claims, 63% had suboptimal maintenance medication adherence (PDC < 80%).
Menzies-Gow et al.	Bulgaria, Canada, Denmark, Greece, Italy, Japan, Kuwait, South Korea, Spain, UK, and USA	2022	Longitudinal	*Asthma patients*	Severe	3531	18–34: Continuers: 11.2%; Stoppers: 15.4%; Switchers: 13.2%; 35–54: Continuers: 38.0%; Stoppers: 34.6%; Switchers: 43.3%; 55–79: Continuers: 48.7%; Stoppers: 47.1%; Switchers: 41.4% ≥ 80: continuers: 2.1%; stoppers: 2.9%; Switchers: 2.1%	Continuers: 1.391 (33.5%)/2.762 (66.5%); Stoppers: 176 (32.7%)/363 (67.3%); Switchers: 192 (33.7%)/377 (66.3%)	omalizumab, mepolizumab, reslizumab, benralizumab, dupilumab	insufficient clinical efficacy; adverse outcomes; High blood eosinophil counts (BEC ≥ 300 cells/µL); FeNO ≥ 50 ppb; Previous use of theophylline as an add-on; More exacerbations in the 12 months prior to biologic initiation; Eosinophilic CRS without nasal polyps	Maintenance oral corticosteroid (OCS) use; Presence of CRSwNP (chronic rhinosinusitis with nasal polyps); Lower disease burden pre-biologic (fewer exacerbations, less HCRU)	“Continuers” = patients who remained on the same biologic for at least 6 months “Switchers” = patients who changed biologic therapy at least once “Stoppers” = patients who discontinued biologic therapy without switching	Continuers: 2791 (79.0%); Stoppers: 356 (10.2%); Switchers: 384 (10.8%)
Corren & Panettieri	USA	2018	Editorial – excluded from empirical synthesis	Severe asthma patients	Severe	-	-	-	Biologics (general)	Poor ICS adherence	Improved ICS adherence before biologics	-	Not applicable
Gelhorn et al.	USA	2019	Cross-sectional	Severe asthma patients and doctors	Severe	47	49.9 ± 13.1	12 (25.5%)/35 (74.5%)	Biologics (general)	Out-of-pocket cost, Complex insurance coverage, Scheduling difficulties, Inconvenient administration site, Needle phobia/injections, Time required for administration, Perceived lack of efficacy	Perceived efficacy, Less frequent administration (preferred every 8 weeks), Subcutaneous (SC) administration, Treatment at specialist clinics, Rapid onset of action	-	Survey (no rate)
Gleeson et al.	USA	2023	Retrospective cohort (1 year)	Patients with asthma	Moderate-to-severe	Total participants: 9,147 adults with asthma; Biologic prescriptions: 306 patients (3.8%); Complete dispensing data: 238 patients; Received at least one dose: 202 patients within 12 months	18–34 years: 1307 (16.2%) 35–44 years: 999 (12.4%) 45–54 years: 1479 (18.4%) 55–64 years: 1928 (23.9%) 65 + years: 2344 (29.1%)​	2,366 (29.4%)/5,691 (70.6%)	Omalizumab, Mepolizumab, Benralizumab, Reslizumab, Dupilumab	Black race (IRR = 0.85) and Medicaid insurance (IRR = 0.86) were independently associated with reduced primary adherence. Patient-level barriers (72.2%) were the leading causes of nonadherence, including: • High out-of-pocket costs • Needle aversion • Lack of follow-up or decision to delay/decline initiation Insurance denial (22.2%) Socioeconomic disparities: Fewer biologic days covered were associated with Medicaid status and Class III obesity. Delays in initiation: Average time from prescription to first dose was 32.5–57.8 days, likely due to logistical or personal barriers rather than system-level delays.	Asthma severity: More oral corticosteroid bursts and recent hospitalizations were strong predictors for receiving a biologic prescription. Nasal polyposis was also positively associated with prescription	(1) primary adherence, or whether a dose of a biologic was received in the 12-month period after initial prescribing, and (2) biologic days covered in the 12-month period after initial prescribing (PDC)	84.9% primary adherence within 12 months
Granda et al.	Spain	2022	retrospective, observational, real-life study	Severe asthma on anti-IL5	Severe	53	61 (51.8–67.0)	33 (61%)/20 (39%)	anti-interleukin-5 biologics	-	-	pharmacy refill data and test	According to the pharmacy refill data lack of adherence to the primary inhaler was 58.5%. Test of Adherence to Inhalers questionnaire was 22.6%. Combining both methods, 17% were considered to have nonadherence
Inselman et al.	USA	2020	Retrospective cohort	Asthma patients	-	2,007,720	Biologic commercial: 41.4 ± 15.0; biologic medicare: 65.5 ± 10.4; non-biologic commercial: 31.4 ± 20.9; non-biologic medicare: 70.2 10.2	888,650/1,118,981	Biologics (general)	High cost of biologics; Socioeconomic and insurance disparities; Limited access in small or non-specialist practices; Reimbursement and drug delivery models (buy-and-bill, white bagging, brown bagging); Prior authorization requirements; Lack of patient awareness	Specialist access (allergist or pulmonologist); Higher income; Private/commercial insurance coverage; Age 18–64 years	-	Peak prevalence of 2.95 per 1,000 asthma patients (omalizumab, in 2006). After 2015, usage of newly approved biologics (e.g., mepolizumab, benralizumab, dupilumab) increased, but remained below historic levels
Ledford et al.	USA	2023	Longitudinal (1 year)	Severe asthma patients	Severe	2117	55 ± 14	699 (33%)/1418 (67%)	Mepolizumab, Benralizumab, Dupilumab, Omalizumab, Reslizumab	Costs, Health insurance-related obstacles, Misunderstanding of instructions Denial of illness, Cultural or religious concerns, Negative perception of treatment, Fear of long-term adverse effects, Stigmatization of treatment	Home options, Less frequent dosing (e.g., benralizumab every 8 weeks), Commercial health insurance (without PCP referral), Shared decision-making regarding administration setting	PDC	The median PDC was 87%; Dupilumab has the lowest adherence rate
Maddux et al.	USA	2021	Retrospective cohort (6 months)	Severe asthma patients	Severe	5319	0–17: 301 (5.66%); 18–64: 4101 (77.10%); > 65: 917 (17.24%)	2,055 (38.6%)/3,264 (61.4%)	Omalizumab, Mepolizumab, Reslizumab, Benralizumab, Dupilumab	-	older age, male sex, Medicare Advantage insurance type, no specialist care, no COPD, depression, and pre-biologic ICS PDC ≥ 0.75	PDC	The mean PDC for asthma biologics was 0.76 (95% CI, 0.75–0.77) in the first 6 months after starting. 61% achieved a biologic PDC ≥ 0.75. Benralizumab: *n* = 141; mean, 80.79 (SD, 17.9); dupilumab: *n* = 251; mean, 98.15 (SD, 6.83); mepolizumab: *n* = 763; mean, 88.49 (SD, 14.19); omalizumab: *n* = 4100; mean, 71.89 (SD, 22.73); and reslizumab: *n* = 64; mean, 92.04 (SD, 9.79)
Osazuwa-Peters et al.	USA	2022	Retrospective cohort (1 year)	Moderate-to-severe asthma patients	Moderate-to-severe	3932 Of whom: *n* = 2,898 (73.7%) Clinic-only subgroup; *n* = 786 (20%) Home subgroup; *n* = 248 (6.3%) Hybrid subgroup	Clinic subgroup: 58.00 [47.00, 68.00]; Home subgroup: 66.00 [52.00, 72.00]; Hybrid subgroup: 68.00 [61.00, 74.00]	1404 (35.71%)/2528 (64.29%)	Omalizumab, Mepolizumab, Reslizumab, Benralizumab, Dupilumab	Black race, Hispanic ethnicity, Lower education, Medicare-only insurance and higher patient out-of-pocket costs	subspecialist access, home setting and hybrid setting, older age, sinusitis and depression, more severe diagnosis	-	Biologic adherence was 0.75 [0.5–1] in the clinic setting (the most common), and 0.83 [0.5–1] in both home and hybrid settings.
Silver et al.	USA	2022	Retrospective cohort	Severe asthma patients	Severe	285	About 45	138 (48.42%)/147 (51.58%)	Not specified	-	no improvement in symptoms (shortness of breath (23.4%), other chronic symptoms (10.9%), ≥ 2 exacerbations in the previous year (10.9%); lack of symptoms control; patients request (46.9%)	-	85 (29.82%) didn’t maintain therapy
Stempel & Szefler	USA	2022	Editorial – excluded from empirical synthesis	Severe asthma patients	Severe	-	-	-	Biologics (general)	Lack of standardized criteria for biologic escalation Socioeconomic and access disparities Patient-level barriers (fear of injections, cost perception) Healthcare system-level barriers (e.g., specialist access, administrative complexity) High cost of biologic medications	-	-	Not applicable
Le et al.	USA	2024	Prospective cohort	Severe asthma patients	Severe	9587 Among patients in the biologics accessible (*n* = 5073), biologics inaccessible (*n* = 3041), and biologics accessible but not received (*n* = 382) groups, *n* = 4691received biologics	54 ± 14.2	Female 2932 (63.0)	omalizumab, mepolizumab, reslizumab, benralizumab, or dupilumab	-	-	-	8.3% non-adherence rate
Crimi et al.	Italy	2023	Cross sectional	Asthma patients	severe	116	-	-	omalizumab, mepolizumab, reslizumab, benralizumab, or dupilumab	Afraid of self-administration	Simplifying procedures and providing more effective and widespread information	Self-report	95% self-reported good adherence and satisfaction with biologics

### Adherence Rates

Adherence rates to biologic therapies for moderate-to-severe asthma vary across studies, depending on study design, adherence assessment methods, and patient characteristics. Most commonly, adherence has been measured using the PDC, which is considered a reliable proxy for treatment continuity and represents the percentage of days within a defined period during which the patient had access to the prescribed medication [18]. Several retrospective U.S.-based studies report generally good, although suboptimal, adherence levels. Ledford et al. reported a median PDC of 87%, with notable differences across individual biologics - Dupilumab showing the lowest adherence [19]. Similarly, Maddux et al. found a mean PDC of 0.76 during the first six months of treatment, with 61% of patients reaching a PDC ≥ 0.75. Dupilumab (98.15%) and Reslizumab (92.04%) had the highest adherence, while omalizumab had the lowest (71.89%) [9].

Osazuwa-Peters et al. also reported favorable adherence rates, with a median PDC of 0.75 in clinic-based settings and 0.83 in home or hybrid settings, suggesting that flexible treatment settings may support higher adherence [20]. In contrast, Bender et al. found that 63% of patients with maintenance medication prescriptions had suboptimal adherence (PDC < 80%), particularly among those with milder disease (Global Initiative for Asthma, GINA steps 2–3) [21].

Primary adherence—defined as the receipt of at least one dose of a prescribed biologic within 12 months—was high in the study by Gleeson et al., at 84.9%. However, only 3.8% of the full adult asthma cohort received a biologic, highlighting access limitations [22]. In a Spanish real-world study, Granda et al. integrated refill data with self-reported measures, reporting a nonadherence rate of 17% to biologic therapy. However, nonadherence to baseline inhaled treatments was notably higher at 58.5%, underscoring the broader challenges faced in asthma management [23].

The large, multinational analysis by Menzies-Gow et al. found that 79% of patients remained on the same biologic for at least six months (“continuers”), while 21% either discontinued (“stoppers”) or switched (“switchers”) biologic treatment [24]. Le et al. reported a low nonadherence rate of 8.3%, though this applied only to patients who had actual access to biologics [25]. High self-reported adherence and treatment satisfaction were also found in the Italian cross-sectional study by Crimi et al., with 95% of patients declaring good adherence. However, reliance on self-report instruments may overestimate true adherence [26].

Other studies provide additional insights. Although Inselman et al. did not report PDC values, they highlighted low overall biologic utilization due to systemic, socioeconomic, and insurance-related factors [27]. Silver et al. found that 29.8% of patients discontinued treatment, often due to perceived lack of efficacy [28]. Editorials by Corren & Panettieri [29] and Stempel & Szefler [30], while lacking quantitative data, emphasize the wide variability in biologic use and persistence, driven by patient-level, economic, and healthcare system barriers.

Overall, the evidence points to substantial heterogeneity in adherence estimates across studies, reflecting differences in study design, settings, biologic agents, and adherence assessment methods. Variability in patient populations—including disease severity, comorbidities, and previous treatment histories—further contributes to the wide range of reported adherence rates.

In the following sections, we report the main barriers and facilitators to adherence identified across the reviewed studies.

### Socioeconomic and System-level Factors

Socioeconomic and healthcare system-related factors constitute a primary influence on adherence to biologic therapies in people with moderate-to-severe asthma. Among the most commonly reported barriers are high out-of-pocket costs, which can impose a significant financial burden and discourage consistent treatment uptake [21, 26, 30]. Several studies have highlighted that patients with lower socioeconomic status or public insurance coverage are at higher risk of suboptimal adherence, partly due to restricted access to specialist care and difficulties in navigating insurance authorizations [19, 21, 26]. Indeed, complex reimbursement policies and administrative requirements, such as prior authorizations, “buy-and-bill” logistics, or “white bagging” delivery models, often delay treatment initiation and complicate continuity of care [18, 26].

Logistical barriers also play a non-negligible role. Difficulties in scheduling appointments, inconvenient administration sites, and delays between prescription and the first dose—ranging from 32 to 58 days—were frequently reported [21, 30]. These factors are particularly relevant in patients who may lack flexible work schedules, transportation, or health literacy needed to navigate such systems. In this context, treatment delivery models that offer at-home or hybrid administration options appear to improve adherence, likely by reducing travel and time burden while enhancing patient autonomy [20]. Similarly, streamlined procedures and a less frequent dosing schedule—such as Benralizumab every eight weeks—have been associated with greater treatment continuity [18, 30].

Conversely, access to private insurance and to asthma specialists (e.g., pulmonologists or allergists) has been consistently identified as a facilitator of adherence [19, 26]. These settings not only provide more consistent access to biologics but also increase the likelihood of structured follow-up and patient education.

### Clinical and Disease-related Factors

Clinical characteristics and disease-related variables play a significant role in influencing adherence to biologic therapy among people with asthma. One of the most consistently reported predictors of lower adherence is the absence of perceived symptom burden or a lack of noticeable improvement during treatment. Individuals who do not experience adequate symptom relief —particularly regarding breathlessness or persistent symptoms— are more prone to discontinue therapy, often by their own initiative [28]. Similarly, the perception that symptoms are under control without treatment has been associated with premature discontinuation [20, 27]. Conversely, individuals with more severe disease profiles-such as frequent exacerbations, increased use of oral corticosteroids, or recent asthma-related hospitalizations- are more likely both to be prescribed biologic and to maintain adherence over time [19, 21].

Comorbid conditions also appear to influence adherence patterns. Depression, for instance, has been negatively associated with persistence in treatment in some studies, potentially due to reduced motivation or self-management capacity [8, 19]. Similarly, class III obesity and comorbid COPD have been linked to lower adherence, potentially due to more complex disease management requirements or reduced treatment responsiveness [8, 21]. In contrast, the presence of nasal polyposis—especially when part of an eosinophilic phenotype— has been correlated with higher adherence, likely reflecting improved perceived efficacy in patients with comorbid upper airway inflammation [24].

Baseline adherence to other controller medications also predicted biologic adherence. Poor adherence to ICS prior to biologic initiation was viewed as a risk factor for low biologic persistence, reinforcing the importance of comprehensive adherence assessment before escalation [29]. Conversely, pre-biologic ICS adherence of ≥ 0.75 was a positive predictor of biologic adherence [9]. Additionally, demographic clinical markers such as older age and male sex showed a modest association with better adherence, suggesting that biologic persistence may be higher in more stable or health-literate patient subgroups [9].

### Psychological Factors

Psychological variables play a crucial, and often underestimated, role in shaping adherence to biologic therapies in people with asthma. Among the most frequently reported psychological barriers are needle phobia and general treatment-related anxiety, which can lead to avoidance or delay in initiating biologic therapy [18, 21, 29, 30]. Fear of injections -especially among patients unaccustomed to regular parenteral treatments- constitutes a significant barrier to both initiation and long-term adherence. Similarly, concerns about long-term adverse effects, skepticism regarding the efficacy of biologics, and negative attitudes toward pharmacological therapies have been shown to diminish patients’ willingness to maintain treatment [18, 27].

Another key factor is denial or minimization of illness, which may lead patients to prematurely discontinue treatment based on the belief that their symptoms are under control or that medication is no longer necessary [18, 27]. This cognitive distortion is especially problematic in chronic conditions such as asthma, where symptom variability may reinforce misconceptions about disease resolution. Additionally, perceived stigmatization associated with the use of biologics—whether due to self-injection, visible treatment routines, or assumptions about disease severity—can further reduce motivation to adhere to therapy [19].

Notably, depression has been identified as a significant psychological barrier, as it can diminish patient motivation, impair self-care behaviors, and increase the risk of treatment discontinuation [9]. Studies have shown that the presence of depression is negatively associated with adherence to biologics, particularly when not adequately addressed in clinical practice [8, 19].

On the other hand, several psychological facilitators of adherence have also been identified. Perceived efficacy of the treatment emerged as a powerful driver of adherence across multiple studies, with patients more likely to continue therapy when they notice clear symptom improvement [25, 30]. Moreover, treatment satisfaction and emotional reassurance, especially when biologics are administered in supportive settings (e.g., specialist clinics), contribute to maintaining long-term engagement with therapy [26]. Importantly, shared decision-making, where patients are actively involved in the choice of treatment modality and setting, has been associated with greater adherence and a more positive therapeutic alliance [19].

**Fig. 2:**
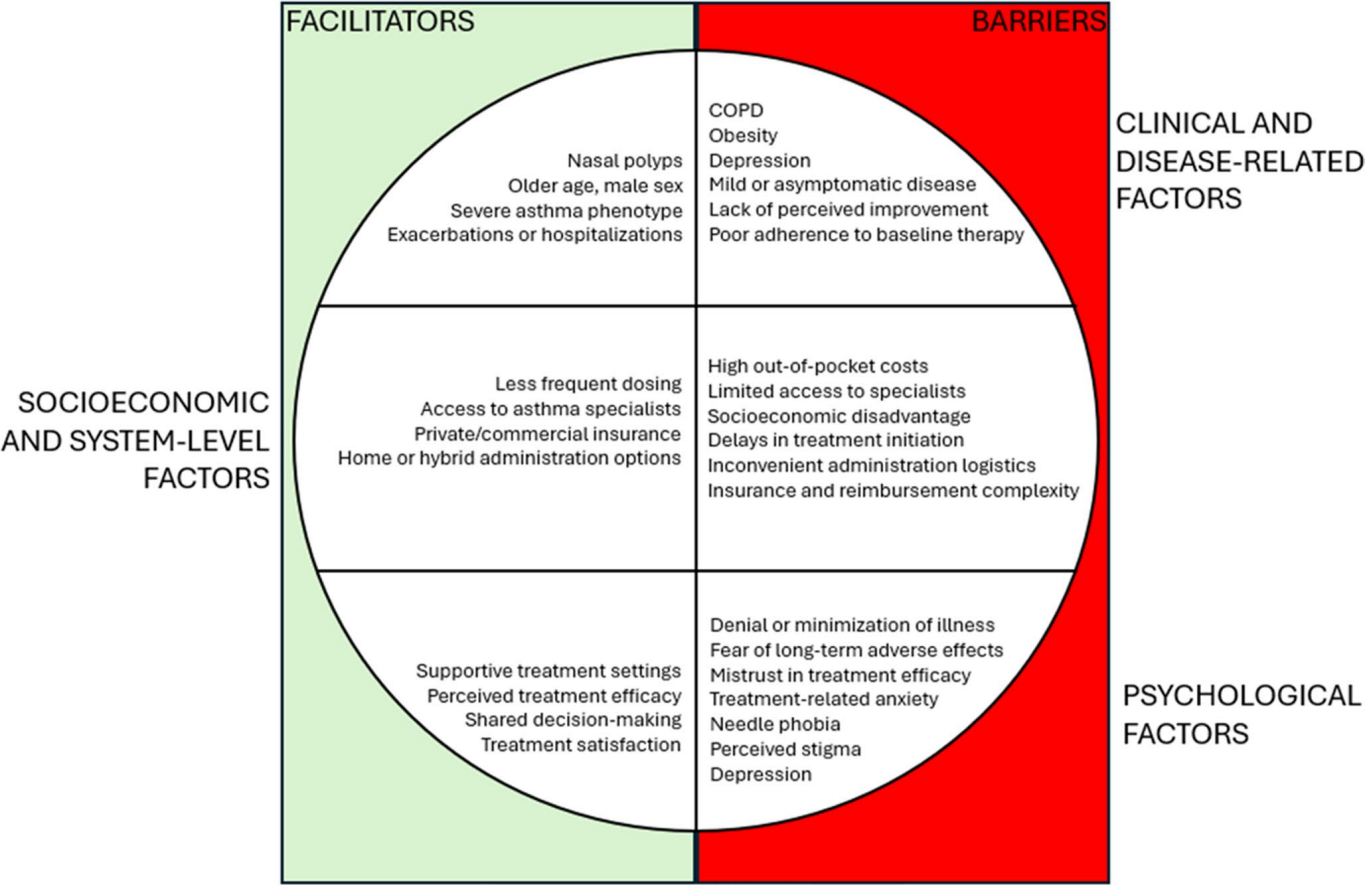
Barriers and facilitators of adherence to biologics in asthmatic patients

Figure 2 shows barriers and facilitators factors related to adherence to biologics.

## Discussion

This review highlights that adherence to biologic therapies in asthma is a multidimensional phenomenon that cannot be fully understood through a purely biomedical lens. While biologics have markedly improved the management of severe asthma, their effectiveness relies on sustained, long-term use, an aspect influenced by far more than pharmacological efficacy alone [32]. The findings of this review illustrate how adherence is shaped by the interplay of systemic, clinical, and psychological factors, underscoring the need for integrated models of care [33].

Adopting a biopsychosocial perspective allows us to capture this complexity. The reviewed literature points to a wide range of system-level influences on adherence, reflecting how structural and organizational constraints shape patients’ real-world ability to initiate and maintain biologic therapy [21, 26, 30]. Rather than functioning as isolated obstacles, these contextual elements tend to accumulate, especially for individuals with limited socioeconomic resources or restricted access to specialized care, creating a structural environment that can meaningfully affect treatment continuation.

However, psychological factors should not be overlooked, as they meaningfully influence adherence behaviors in patients with asthma. Beyond clinical outcomes alone, patients’ interpretations of their symptoms, expectations of improvement, and beliefs about disease control contribute to decisions about continuing or discontinuing treatment [27]. Comorbid psychological conditions—particularly depression—may further reduce motivation and compromise engagement, yet they remain insufficiently assessed and addressed within routine asthma care [9].

Psychological mechanisms—including treatment-related anxiety, illness denial, and stigma—highlight that adherence is shaped not only by concerns about medications but also by the emotional and symbolic meanings patients attribute to biologic therapy [34]. These experiences, often unspoken during clinical encounters, can have a substantial impact on treatment behavior.

Importantly, the review also identified facilitators of adherence, such as perceived treatment benefit, emotional reassurance, and collaborative decision-making processes [19]. These findings reinforce that the therapeutic relationship, communication quality, and patient involvement are not ancillary aspects of care but central determinants of long-term engagement. Treatment settings perceived as supportive—whether in specialist clinics or structured home-care models—can further enhance adherence by reducing psychological and organizational burden.

Together, these findings suggest that clinicians must go beyond the prescription. Understanding how patients make sense of their illness, what meanings they assign to biologic therapies, and how they emotionally and cognitively respond to the idea of long-term treatment is essential. Adherence is not merely a matter of instruction and compliance—it is embedded in the patient’s subjective experience and day-to-day reality [28]. Recognizing this dimension can help clinicians identify vulnerabilities, tailor interventions, and better support sustained engagement with biologic therapy.

### Strengths and Limitations

This scoping review offers an integrative synthesis of psychological and related biopsychosocial factors influencing adherence to biologic therapies in asthma—a topic that has received limited attention to date. By systematically mapping existing evidence, the review highlights converging findings across disciplines and identifies key gaps to inform future research and practice.

Nonetheless, several limitations should be acknowledged. The search was restricted to peer-reviewed articles and excluded grey literature, which may have introduced publication bias. Most included studies were observational, conducted in Western countries, and used heterogeneous definitions and measures of adherence, limiting direct comparability. Furthermore, psychological determinants were often examined as secondary outcomes, precluding quantitative synthesis. Despite these constraints, this review provides a timely and comprehensive overview that advances understanding of a complex and understudied issue.

### Clinical Implications

From a clinical standpoint, this scoping review calls for a paradigm shift in asthma care. Beyond assessing biological eligibility (e.g., eosinophil counts, exacerbation history), it is crucial to evaluate psychological readiness, health beliefs, emotional distress, and contextual challenges. For example, a patient struggling with anxiety or low mood may benefit from brief psychological interventions, such as cognitive-behavioral strategies or motivational interviewing, delivered in collaboration with mental health professionals [35]. Similarly, those facing systemic barriers may require case management, social support, or telehealth options that adapt treatment to real-world constraints [36].

Clinically, this can be operationalized using validated screening tools for psychological distress and barriers to adherence, training in empathic communication, and a multidisciplinary approach involving pulmonologists, psychologists, nurses, and care navigators. These interventions need not be resource-intensive; even brief, structured actions integrated into routine care can significantly improve adherence and therapeutic alliance.

Finally, this model aligns with the broader concept of precision medicine [37]—not only at the molecular or biological level, but also in recognizing that patient care must be tailored to psychological, relational, and environmental dimensions. The success of biologic therapies depends as much on the human context as on the molecule itself. Promoting patient empowerment, fostering a collaborative therapeutic relationship, and respecting individual variability are essential for optimizing outcomes in chronic and emotionally demanding conditions such as severe asthma [38].

### Future Directions

Future studies should further explore the psychological mechanisms underlying adherence to biologic therapies, such as expectations, emotional regulation, and treatment-related beliefs. Longitudinal and mixed-methods designs could clarify causal pathways and temporal changes in adherence behaviors. Additionally, intervention studies integrating behavioral science and patient-centered approaches are needed to develop tailored strategies that enhance long-term engagement with biologic treatments. Collaborative efforts between clinicians, psychologists, and healthcare systems will be critical to translate these insights into routine asthma care.

## Conclusion

Evidence from this scoping review suggests that adherence to biologic therapies in asthma is shaped by psychological, contextual, and systemic factors that extend beyond pharmacological efficacy. Supporting patients through integrated, person-centered approaches may improve long-term treatment engagement. Future studies should further clarify these mechanisms and develop tailored interventions that align clinical goals with patients’ lived experiences, ultimately improving both adherence and quality of life.

### Key References

- Menzies-Gow AN, McBrien C, Unni B, Porsbjerg CM, Al-Ahmad M, Ambrose CS, et al. Real world biologic use and switch patterns in severe asthma: data from the international severe asthma registry and the US CHRONICLE study. J Asthma Allergy. 2022;15:63–78. 10.2147/JAA.S328653.
  - ○ This large, multinational study provides essential real-world data on biologic use, persistence, and switching patterns in severe asthma, highlighting key clinical and systemic factors that influence adherence.
- Silver J, Bogart M, Molfino NA, Siddall J, Small M, Hanson M, et al. Factors leading to discontinuation of biologic therapy in patients with severe asthma. J Asthma. 2022;59:1839–49. 10.1080/02770903.2021.1971700.
  - ○ This paper identifies patient-reported reasons for discontinuing biologic treatment, emphasizing the importance of perceived efficacy, psychological readiness, and shared decision-making in sustaining adherence.
- Le TT, Price DB, Erhard C, Cook B, Quinton A, Katial R, et al. Disease burden and access to biologic therapy in patients with severe asthma, 2017–2022: an analysis of the international severe asthma registry. J Asthma Allergy. 2024;17:1055–69. 10.2147/JAA.S468068.
  - ○ This recent international analysis underscores ongoing disparities in access to biologics and illustrates how socioeconomic and health-system barriers continue to shape real-world adherence and outcomes.

## Supplementary Information

Below is the link to the electronic supplementary material.

Supplementary File 1 (DOCX 14.9 KB)

## Data Availability

The authors confirm that the data supporting the findings of this study are available within the article and its supplementary materials.
