# Supplementary material for: Psychological and Related Factors Influencing Adherence to Biologic Therapies in Asthma: A Scoping Review
Source: Curr Allergy Asthma Rep. 2025 Dec 17;25(1):60. doi: 10.1007/s11882-025-01242-5 (PMC12711935; doi:10.1007/s11882-025-01242-5)
Supplement: Supplementary file 1 — (DOCX 14.9 KB) [file 11882_2025_1242_MOESM1_ESM.docx]

| **Database** | **Key words** | **Filters applied** |
| --- | --- | --- |
| PubMed | ((asthma AND biologics) AND (adherence AND adherence to treatment)) | Species: Humans;  Other: Exclude Preprints |
| Scopus | Asthma AND biologics AND adherence AND adherence to treatment | Publication stage: Final;  Language: English |
| APA Psycnet | Asthma AND biologics AND adherence AND adherence to treatment | No filter added |

**Additional File 1.** Search strategy
